# Nanocrystalline TiO_2_ Composite Films for the Photodegradation of Formaldehyde and Oxytetracycline under Visible Light Irradiation

**DOI:** 10.3390/molecules22060950

**Published:** 2017-06-14

**Authors:** Min Wei, Xue-Lei Peng, Qi-Sheng Liu, Fang Li, Ming-Ming Yao

**Affiliations:** 1 Key Laboratory of Interfacial Reaction & Sensing Analysis in Universities of Shandong, School of Chemistry and Chemical Engineering, University of Jinan, Jinan 250022, China; emily518@foxmail.com (M.W.); chm_liuqs@ujn.edu.cn (Q.-S.L.); chm_lif@ujn.edu.cn (F.L.); 2 Jinan Institute of Product Quality Inspections, Jinan 250022, China; yaomm4364@sina.com

**Keywords:** TiO_2_ film, multi-modification, photodegradation

## Abstract

In order to effectively photodegradate organic pollutants, ZnO composite and Co-B codoped TiO_2_ films were successfully deposited on glass substrates via a modified sol-gel method and a controllable dip-coating technique. Combining with UV–Vis diffuse reflectance spectroscopy (DRS) and photoluminescence spectra (PL) analyses, the multi-modification could not only extend the optical response of TiO_2_ to visible light region but also decrease the recombination rate of electron-hole pairs. XRD results revealed that the multi-modified TiO_2_ film had an anatase-brookite biphase heterostructure. FE-SEM results indicated that the multi-modified TiO_2_ film without cracks was composed of smaller round-like nanoparticles compared to pure TiO_2_. BET surface area results showed that the specific surface area of pure TiO_2_ and the multi-modified TiO_2_ sample was 47.8 and 115.8 m^2^/g, respectively. By degradation of formaldehyde and oxytetracycline, experimental results showed that the multi-modified TiO_2_ film had excellent photodegradation performance under visible light irradiation.

## 1. Introduction

With industrialization and population growth, water pollution caused by recalcitrant organic compounds is becoming a major environmental problem [1]. In recent years, traditional physical and biological treatment methods—such as activated carbon adsorption, ultra-filtration, reverse osmosis, and coagulation—are used to remove the contaminants from wastewater. However, these techniques cannot transfer organic compounds into non-hazardous compounds [2]. Semiconductor heterogeneous photocatalysis, a new water treatment technology, is considered as an effective environmentally-friendly approach to decrease the concentrations of organic contaminants in various wastewaters [3]. Among various semiconductors, titanium dioxide is considered to be the most useful photocatalyst due to its physical and chemical stability, photo-corrosion resistance, non-toxicity, convenience of preparation, cost-effective breakdown of harmful organic molecules, etc. [4]. Powdered TiO_2_ is limited as a photocatalyst since a post treatment separation is required to recover its photocatalysis in wastewater. Therefore, TiO_2_ films coated on various substrates such as glass and ceramic tiles have attracted considerable interests recently [5].

According to our knowledge, anatase and rutile forms of TiO_2_ have been investigated extensively as photocatalyst among the three common crystalline forms (anatase, brookite, and rutile), and antase-TiO_2_ possess the best photocatalytic activity [6]. Since the light response range of pure TiO_2_ is limited in the UV-light region, only photons with energies equal or greater than the band gap energy (ΔE ≈ 3.2 eV) can generate positive electrons (e^−^) and holes (h^+^) which then promote possible photocatalytic reactions [7]. When the generated charge carriers react with oxygen/water neighboring the photocatalyst, hydroxyl radicals with strong oxidation can be generated. In the light of these highly active radicals, the degradation process of organic pollutants can be easily caused in the air and water solution. This is the reason why TiO_2_ can completely oxidize large quantities of aquatic organic pollutants to CO_2_ and H_2_O through both oxidative degradation and reductive transformation. Unfortunately, the practical application of TiO_2_ in photocatalysis is limited by its lower efficiency, which boils down to (i) an increase of the electron-hole pairs recombination rate i.e., a decrease of their lifetime; and (ii) the wide band-gap restricting light absorption to only ultraviolet region, thus limiting the range of light response to visible light region. To overcome the inherent weakness of TiO_2_, numerous efforts have been made to increase its visible light absorption and prolong carrier’s lifetime. Many popular techniques are used to modify TiO_2_ including composite semiconductors, metal doping, and nonmetal doping [8,9].

**Figure 1:**
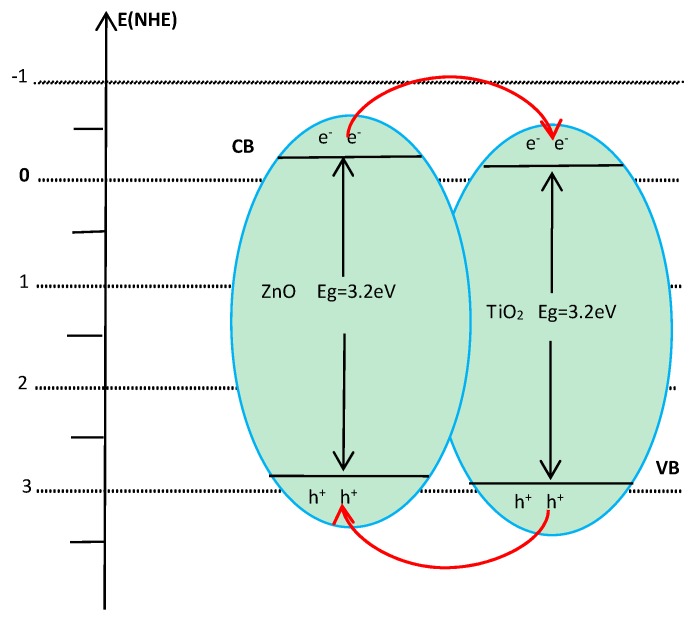
Schematic diagram for charge transfer in composite TiO_2_/ZnO film.

Coupling with another semiconductor is regarded as a good method to improve the photocatalytic activity of TiO_2_. Reportedly, ZnO is an excellent n-type semiconductor material exhibiting promising efficiency for photocatalytic oxidation of organic contamination. It is due to the intimate interfacial interaction between ZnO and TiO_2_ via chemical bonding that ZnO can uniformly graft on the surface of TiO_2_ to form the heterojunction. The mechanism for charge transfer in composite TiO_2_-ZnO film can be graphically shown in Figure 1. The slight negative shift of the ZnO band facilitates the injection of electrons from the CB of ZnO to the CB of TiO_2_, and the migration of holes from the VB of TiO_2_ to the VB of ZnO upon illumination, which effectively increase the availability of e^−^ and h^+^ for targeted redox processes by reducing their recombination [10]. The technique may improve the photocatalytic performance of TiO_2_ in a certain extent, but still cannot meet the actual needs. In order to effectively photodegrade organic pollutants, other modification methods also need to be used.

Metal doping is another effective approach to modify TiO_2_ films. It was commonly reported that Co doping can enhance the photocatalytic activity of TiO_2_ due to narrowed band gap and diminished photo-generated electrons and holes combination [11], but difficulties involved in metal doping into TiO_2_ are poor thermal stability during heat treatment, surface aggregation rather than substitution, and only a small red shift [12]. It is accepted that doping with nonmetal ions into a titania matrix provides favorable visible light response and facilitates efficient charge carrier transfer processes. B ions can be mainly incorporated into some metal oxide lattices in interstitial mode acting as shallow traps for electrons to prolong the life of the photo-generated electrons and holes [13]. Normally, metal dopant energy levels are below the conduction band edge of TiO_2_, while nonmetal dopant energy levels are just above the top of the valence band of TiO_2_. This gives us a hint that low concentration codopants of metal and nonmetal ions may effectively narrow the band gap and enhance the visible light absorption efficiency. The aim of this study is to examine the effect of ZnO composite and Co-B codoping for TiO_2_ photocatalysts on the photocatalytic degradation of oxytetracycline (OTC) and formaldehyde in an aqueous solution. It is expected that the multi-modified TiO_2_ film can exhibit better photocatalytic properties due to the synergistic effect of two processes.

## 2. Experimental

### 2.1. Film Preparation

**Figure 2:**
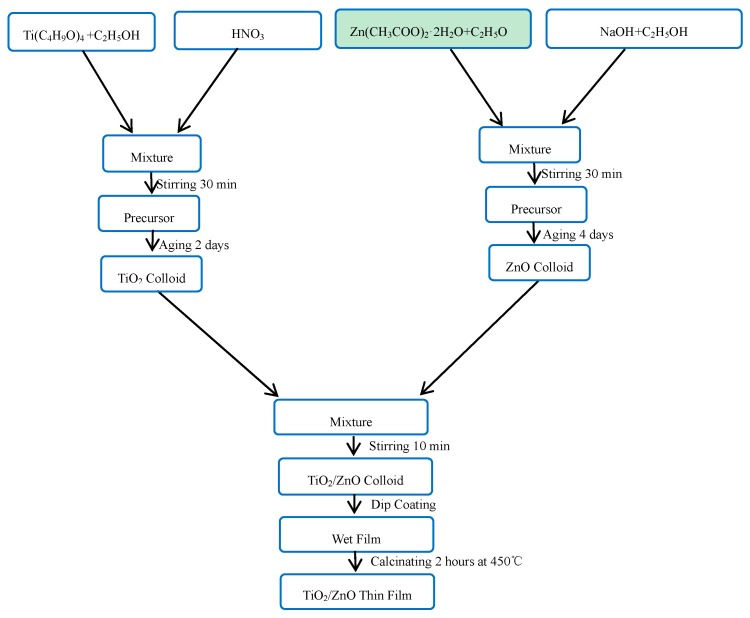
Flow chart of composite TiO_2_/ZnO film preparation.

All reagents and chemicals were of analytical grade without any further purification and the water used was double-distilled deionized, invariably, in our experiment. Pure TiO_2_, composite TiO_2_/ZnO, Co doped TiO_2_/ZnO, B doped TiO_2_/ZnO, and Co-B codoped TiO_2_/ZnO films were prepared via applicable sol-gel technique employing tetrabutyl titanate (Ti(C_4_H_9_O)_4_) and zinc acetate dihydrate (Zn(CH_3_COO)_2_·2H_2_O) as metal sources. The TiO_2_ sol was prepared using tetrabutyl titanate, ethanol absolute (solvent), nitric acid (0.2 mol/L, catalyst) in a volume ratio of 1:20:20, correspondingly, in a clean and dry vessel at ambient temperature. The process was completed with vigorous stirring about 30 min till the homogeneous colloidal suspension was obtained, and the preparation procedure is described in detail in reference [14]. The ZnO sol was prepared as follows: the first step is to prepare 5 × 10^−3^ mol/L zinc acetate ethanol solution, in which a certain amount of zinc acetate was dissolved in anhydrous ethanol under vigorous stirring. Then, in a volume ratio of 1:9, 2 × 10^−2^ mol/L sodium hydroxide ethanol solution was added to the above solution dropwise with magnetic stirring for 30 min at room temperature. A transparent ZnO-sol was obtained after for two days [15]. The flow chart of TiO_2_/ZnO composite film preparation is shown in Figure 2.

The targeted films were immobilized to the surface of glass substrate (25 × 25 × 1 mm^3^), which were cleaned by chromic acid lotion, and then rinsed by distilled water and ethanol, using a controllable dip-coating technique at ambient atmosphere. The speed withdrawal was maintained at 1 mm/s. TiO_2_/ZnO composite films were prepared by repeating the deposition procedures of TiO_2_ colloid and ZnO colloid, alternately. 0.1 mL 7 × 10^−3^ M cobalt or 0.1 mL 9 × 10^−2^ M boron ions were doped into the surface layer via coating cobaltous nitrate hexahydrate (Co(NO_3_)_2_·6H_2_O) or boric acid (H_3_BO_3_) aqueous solution onto a dried TiO_2_/ZnO composite film. At last, to remove organic substances contained in the gel and induce crystallization of particles, all as-prepared samples were calcined at 450 °C in a horizontal furnace for 1 h. In our experiments, the thickness of the films was 0.1–0.3 μm measured using a profilometer.

### 2.2. Catalyst Characterization

The UV–Vis diffuse reflectance spectra (DRS) of various films were recorded to analyze the light absorption via a UV–Vis spectrophotometer (TU-1901) equipped with an integrating sphere accessory (IS 19-1) using blank glass plate as a reference. In order to study the recombination of photo-generated electron-hole pairs in the Co-B codoped TiO_2_/ZnO film, a FLS 920 spectrometer, which employed a 300 nm line of 450 W xenon lamps as excitation source, recorded the photoluminescence (PL) emission spectra. The emission was scanned in the region of 300–700 nm. Meanwhile, the widths of both the excitation slit and the emission slit were set to 3.0 and 2.0 nm, respectively. The identity of crystalline phase of the samples was identified by the X-ray diffraction (XRD) patterns, which were obtained from a diffractometer (type DX-2500) employing Cu Kα radiation at a scan rate (2θ) of 0.05° s^−1^, an accelerating voltage of 40 kV and applied current of 25 mA. It is SUPRA 55 high-resolution field emission scanning electron microscope (FE-SEM) that was used to characterize the surface morphology of the samples. X-ray photoelectron spectroscopy (Amicus) analysis was performed with a spectrometer. Charge correction was performed by referencing the C 1s peak for hydrocarbons to a binding energy of 284.8 eV. N_2_ adsorption–desorption isotherms, which were obtained on a ASAP 2020 apparatus, of the samples to analyze the Brunauer–Emmett–Teller (BET) surface area using multipoint BET method.

### 2.3. Catalyst Test

The photocatalytic properties of pure TiO_2_, TiO_2_/ZnO, and Co or B doped TiO_2_/ZnO films were evaluated by degradation of oxytetracycline (5 mg/L) or formaldehyde (5 mg/L) in an aqueous solution under visible light irradiation. Prior to our experimentation, the solution of 5 mL target pollutants in the weighing bottle and the films at the bottom of the bottle were placed in the dark for 30 min before illumination to establish adsorption-desorption equilibrium. To perform the photocatalytic reaction, the pure TiO_2_ and modified TiO_2_ films were exposed to a light source which was positioned over samples at the height of 15 cm. The visible light source came from a tungsten halogen lamp equipped with a UV cut-off filters (λ > 400 nm), whose the average light intensity was 40 mW/cm^2^. UV–Vis Spectrometer (TU-1901) was adopted to assess the photo-degradation activity of the film photocatalysts. The degradation rate of organic pollutants could be calculated by the formula: η = (1 − *c/c*_0_) × 100%, where *c*_0_ is the initial concentration of the organic solutions, while *c* is the final concentration after illumination. In order to detect the concentration of formaldehyde whose colorlessness caused the difficulty of detection, acetylacetone spectrophotometry was selected to determine its content because of its fewer disturbance factors, easy of operation, and good reproducibility. In our experiment, the experimental error was found to be within the acceptable limit (±5%).

## 3. Results and Discussion

### 3.1. Photocatalytic Activity

**Figure 3:**
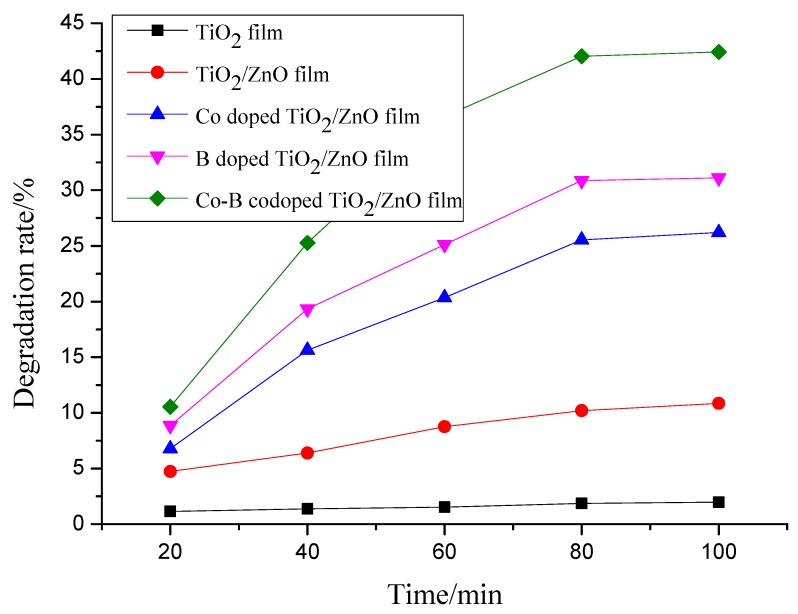
Decomposition kinetics of oxytetracycline solutions using pure TiO_2_, composite TiO_2_/ZnO, Co doped TiO_2_/ZnO, B doped TiO_2_/ZnO, and Co-B codoped TiO_2_/ZnO films on glass substrates under visible light irradiation for 100 min.

Oxytetracycline as an antibiotic is a representative degradation-resistant organic pollutant which can be frequently detected in wastewater, so it is essential to remove it from contaminated water before discharging it into the environment [16]. Figure 3 shows decomposition kinetics of oxytetracycline solutions using pure TiO_2_, composite TiO_2_/ZnO, Co doped TiO_2_/ZnO, B doped TiO_2_/ZnO, and Co-B codoped TiO_2_/ZnO films on glass substrates under visible light irradiation for 100 min. There is almost no degradation for oxytetracycline solutions using pure TiO_2_ film as shown in Figure 3. The degradation percentage of oxytetracycline solutions using Co-B codoped TiO_2_/ZnO film is about 42% at the end of the test, compared with using B doped TiO_2_/ZnO 31%, Co doped TiO_2_/ZnO 26%, and TiO_2_/ZnO 10%, respectively.

**Figure 4:**
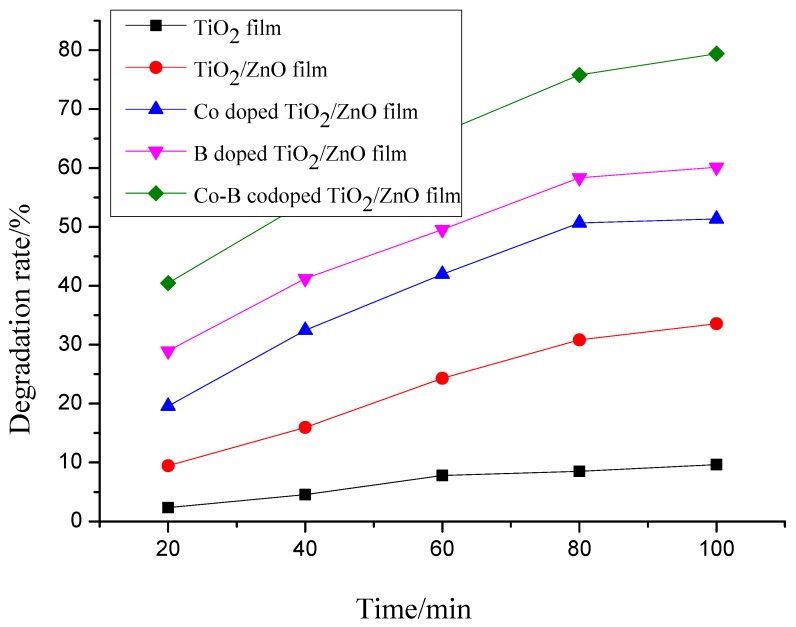
Decomposition kinetics of formaldehyde solutions using pure TiO_2_, composite TiO_2_/ZnO, Co doped TiO_2_/ZnO, B doped TiO_2_/ZnO, and Co-B codoped TiO_2_/ZnO films on glass substrates under visible light irradiation for 100 min.

Formaldehyde as a carcinogen is an organic pollutants which can be also frequently detected in wastewater [17]. Figure 4 shows decomposition kinetics of formaldehyde solutions using pure TiO_2_, composite TiO_2_/ZnO, Co doped TiO_2_/ZnO, B doped TiO_2_/ZnO, and Co-B codoped TiO_2_/ZnO films on glass substrates under visible light irradiation for 100 min. Compared with pure TiO_2_ film 9%, the degradation percentage of formaldehyde solutions using TiO_2_/ZnO, Co doped TiO_2_/ZnO, B doped TiO_2_/ZnO, and Co-B codoped TiO_2_/ZnO film has reached 34%, 51%, 60% and 80%, respectively, during use time in our test. Therefore, the prepared Co-B codoped TiO_2_/ZnO film is effective for the decomposition of both formaldehyde and oxytetracycline solutions under visible light irradiation. The oxytetracycline solution is more difficult due to its stable naphthacene ring structure compared to the degradation for formaldehyde.

From the above experimental results, the combination of TIO_2_ with ZnO and Co/B codoping is an effective way to improve the photocatalytic efficiency of TiO_2_. On one hand, the slight negative shift of the ZnO band can facilitate the injection of electrons from the CB of ZnO to the CB of TiO_2_, effectively increases the availability of e^−^ and h^+^ by reducing their recombination. On the other hand, Co doping can narrow the band gap and enhance the intensity of absorption in the visible region, while B ions can be mainly incorporated into metal oxide lattices acting as shallow traps for electrons to prolong the life of the photo-generated electrons and holes. It is worth noting that the photocatalytic activity of the modified TiO_2_ film is strongly dependent on the doped concentration since the ion doping can serve not only as a mediator of interfacial charge transfer but also as a recombination center. In our experiment, the optimal doped concentrations of Co and B ions were 7 × 10^−3^ and 9 × 10^−2^ mol/L, respectively.

### 3.2. Sample Characterization

#### 3.2.1. Optical Absorption

**Figure 5:**
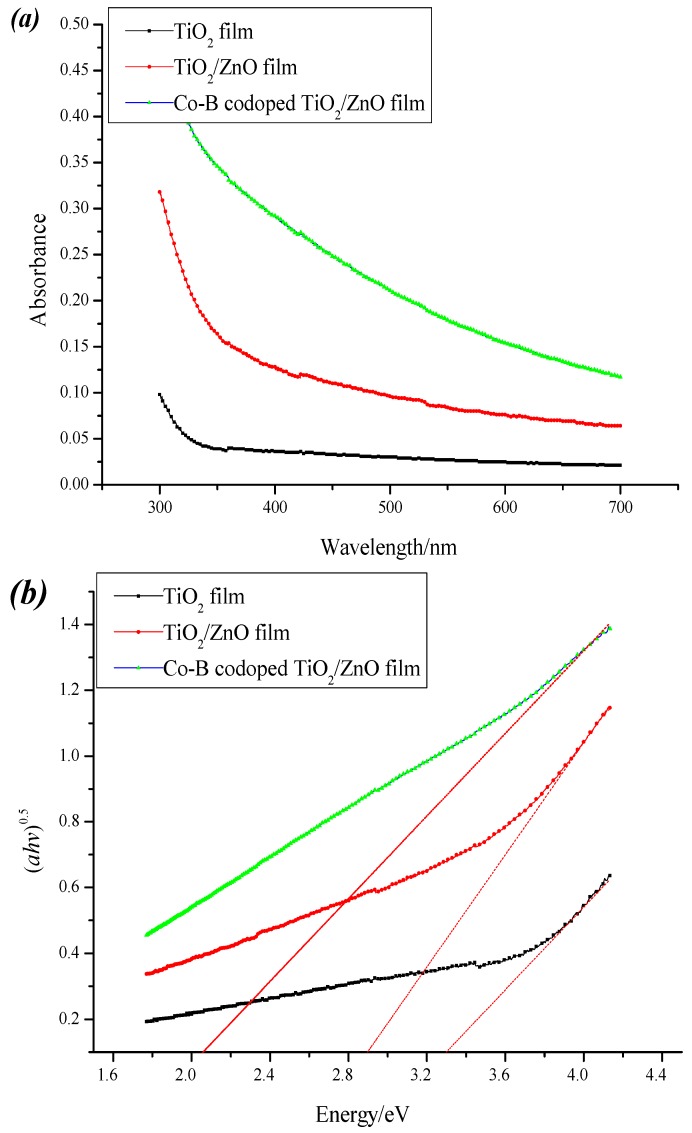
UV–Vis absorption spectra (**a**) and the band gap (**b**) of pure TiO_2_, composite TiO_2_/ZnO, and Co-B codoped TiO_2_/ZnO films.

It is band gap energy that plays vital role in enhancing spectral response to visible region of semiconductor materials. UV–Vis absorption spectra (a) and the band gap (b) of pure TiO_2_, composite TiO_2_/ZnO, and optimal Co-B codoped TiO_2_/ZnO films are depicted in Figure 5. Obviously, the Co-B codoped TiO_2_/ZnO film shows a red shift of the absorption edge to visible light region. The optical band gaps of a crystalline semiconductor can be calculated from the equation: *αhν = A (hν-E_g_)^n/2^*, in which *α*, *h*, E_g_, and A are the absorption coefficient, light frequency, band gap, and a constant, respectively. The value of *n* is determined by the type of optical transition of a semiconductor. The band gap energy of samples can be determined by extrapolation of the liner portion of the (*αhν*)^1/2^ versus energy (*hν*) curve to the energy axis [18]. The calculated energy band gaps (E_g_) of TiO_2_, TiO_2_/ZnO, and Co-B codoped TiO_2_/ZnO films are 3.2, 2.88, and 2.06 eV, respectively. The decrease of the band gap energy is expected to be helpful for the photoactivity because more photoexcited carriers can participate in the photocatalytic reaction and their lifetime is elongated. In other words, the synergetic effects of ZnO composite and Co-B codoping can result in formation of doping level within the band gap of TiO_2_ and thus effectively narrow the band gap.

#### 3.2.2. PL Analysis

**Figure 6:**
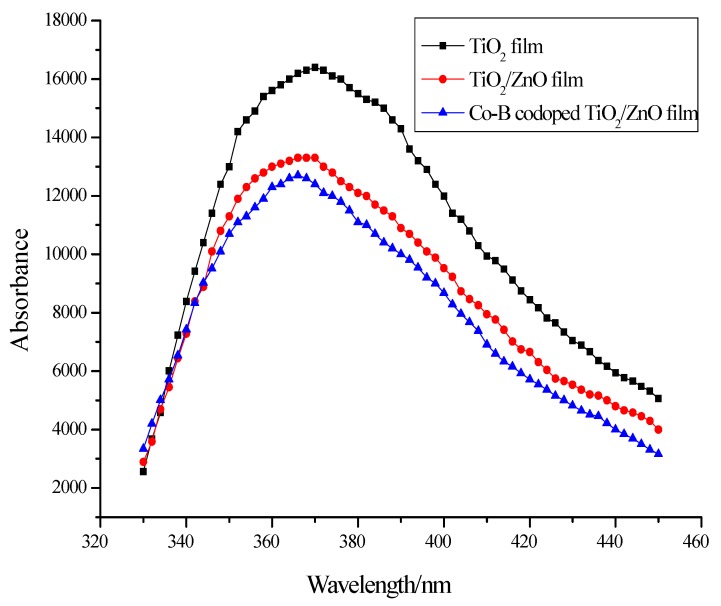
PL spectra of pure TiO_2_, composite TiO_2_/ZnO, and Co-B codoped TiO_2_/ZnO films.

In order to further disclose the effects of ZnO coupling and Co-B codoping on the formation of photoexcited charge carriers and their recombination kinetics, PL spectra are employed to follow the lifetime of photogenerated electron-hole pairs. Figure 6 shows PL spectra of pure TiO_2_, composite TiO_2_/ZnO, and Co-B codoped TiO_2_/ZnO films. Although the peak position (about 370 nm) of the three samples is similar, their PL intensities are quite different. The PL intensity of Co-B codoped TiO_2_/ZnO film is the lowest compared to pure TiO_2_ and composite TiO_2_/ZnO, indicating the recombination of electrons and holes was effectively prohibited. The increased lifetime of the charge carriers can enhance the photocatalytic activity of Co-B codoped TiO_2_/ZnO film.

According to the above analyses, we can draw the conclusion that ZnO coupling and Co-B codoping can not only induce strong visible light absorption but also decrease the recombination rate of electron-hole pairs. It was also noteworthy that Co-B codoping may play a leading role for narrowing the band gap, whereas ZnO coupling may exhibit prior function in prolonging the life time of photoexcited charge carriers.

#### 3.2.3. Crystal Structure

**Figure 7:**
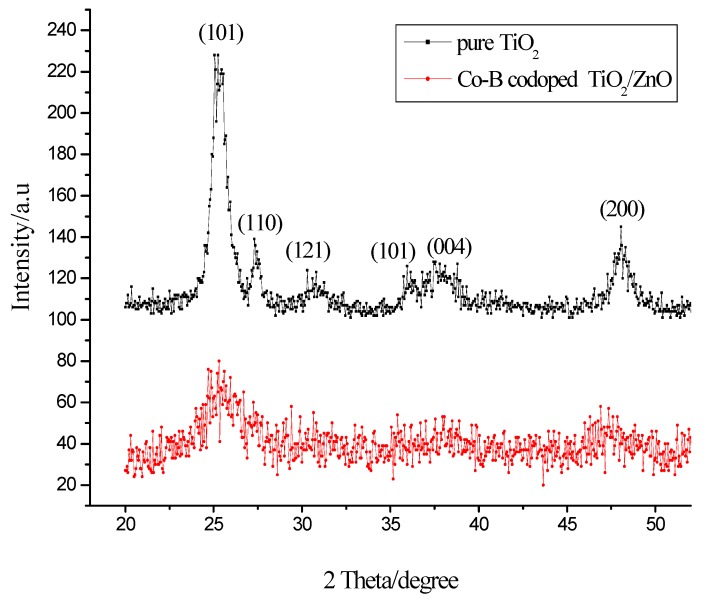
XRD patterns of pure TiO_2_ and Co-B codoped TiO_2_/ZnO samples.

Photocatalytic efficiency of semiconductor materials is strongly dependent on their crystal structure. Figure 7 presents the XRD patterns of pure TiO_2_ and Co-B codoped TiO_2_/ZnO samples calcined at 450 °C in air for 1 h. From Figure 7, pure TiO_2_ shows a mixed crystallinity composed of anatase, brookite, and rutile. The main diffraction peaks at 2θ = 25.3°, 37.3°, and 48.1° are assigned to the (101), (004), and (200) planes of the anatase phase of TiO_2_, respectively. The diffraction peak at 2θ = 30.3° is assigned to the (121) plane of the brookite phase of TiO_2_. The diffraction peaks at 2θ = 27.3° and 36.8° are assigned to the (110) and (101) planes of the rutile phase of TiO_2_. It is clear that the (101) reflections are dominant in the spectrum, well according to the standard diffraction data of TiO_2_ powder (JCPDS No. 21-1276). Compared to the diffraction peaks of pure TiO_2_, those of the Co-B codoped TiO_2_/ZnO are more broad and weak, indicating a small crystal size of this sample [3,19]. According to the Scherrer equation: D = 0.89λ/βcosθ, the average crystallite sizes of pure TiO_2_ and Co-B codoped TiO_2_/ZnO are 7 and 3 nm, respectively, indicating that the multi-modification TiO_2_ can effectively hinder the increase of the crystallite size. In addition, it can be seen that the rutile peaks almost disappear thus forming an anatase-brookite heterojunction. As the latest report, the biphase heterojunction TiO_2_ can achieve higher photocatalytic activity than whichever single phase TiO_2_ due to the synergistic effect between anatase and brookite [20]. The peaks of ZnO are so weak that they can be hardly observed due to two possible reasons. One is that the coupling amount of ZnO in this system is very small. The other is that the peaks of TiO_2_ are so strong that the weak peaks of ZnO are covered [21]. Similarly, there is no Co and B related compound peaks are observed indicating that the small amounts of Co and B dopants are uniformly distributed on TiO_2_.

#### 3.2.4. Surface Morphology

**Figure 8:**
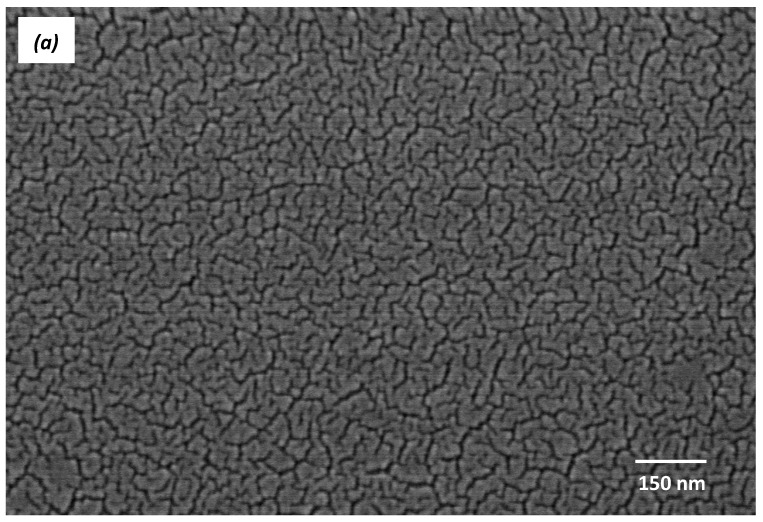

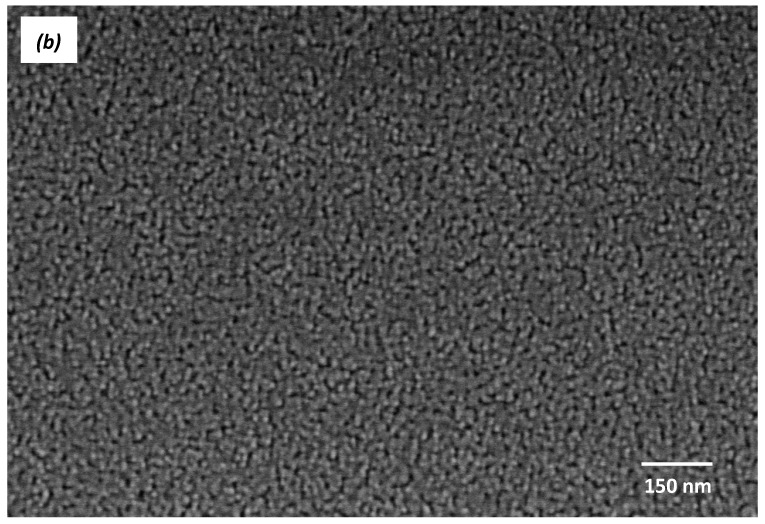
FE-SEM images of (**a**) pure TiO_2_ and (**b**) Co-B codoped TiO_2_/ZnO films.

As we know, the photocatalytic properties of catalysts are usually influenced by their surface morphology. Figure 8 shows FE-SEM micrographs of pure TiO_2_ and Co-B codoped TiO_2_/ZnO films calcined at 450 °C in air for 1 h. Compared with pure TiO_2_ film with cracks, the Co-B codoped TiO_2_/ZnO film without cracks was composed of smaller round-like nanoparticles. It is well-known that a good dispersion or reduced aggregation among particles may increase the active site, thus improving photocatalytic degradation of organic pollutants.

#### 3.2.5. XPS Study

**Figure 9:**
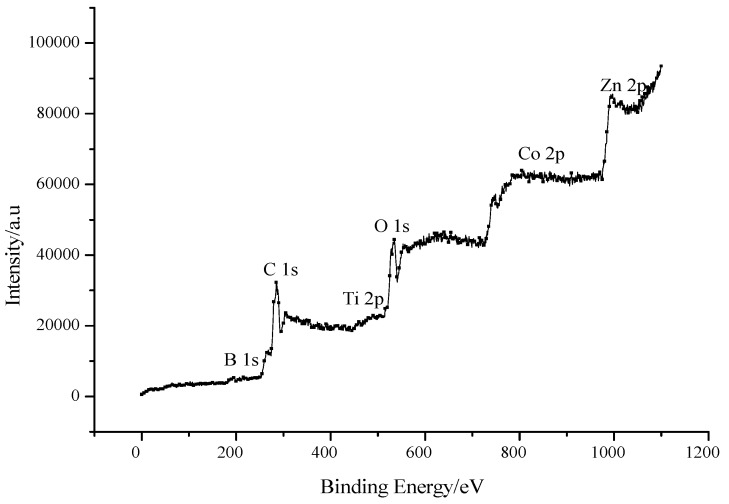
XPS image of Co-B codoped TiO_2_/ZnO film.

X-ray photoelectron spectroscopy can be carried out to determine the surface elemental composition of the catalyst. Figure 9 shows XPS image of Co-B codoped TiO_2_/ZnO film calcined at 450 °C for 1 h. From Figure 9, the characteristic peaks of B 1s, C 1s, Ti 2p, O 1s, Co 2p, and Zn 2p are observed with the binding energies of ~195, 285, 459, 531, 799, and 1044 eV, respectively, consistent with the values reported in literature [22,23]. The presence of C element may come from residual carbon of organic precursors used in the sol-gel method and adventitious hydrocarbon. A weak broad peak with a binding energy centered at ~195 eV is observed for the sample, indicating that the B species may mainly occupy the interstitial site of TiO_2_. In addition, it is found that the incorporation of Co species also facilitates the formation of oxygen vacancies, which may be due to charge imbalance associated with the substitution of Ti^4+^ ions. Composition analysis by energy-dispersive spectroscopy (EDS) indicates that the atomic concentrations of Ti, O, Zn, Co, B, and C in the film are 15.12, 64.90, 0.62, 0.47, 0.79, and 18.10%, respectively.

#### 3.2.6. Surface Areas

**Figure 10:**
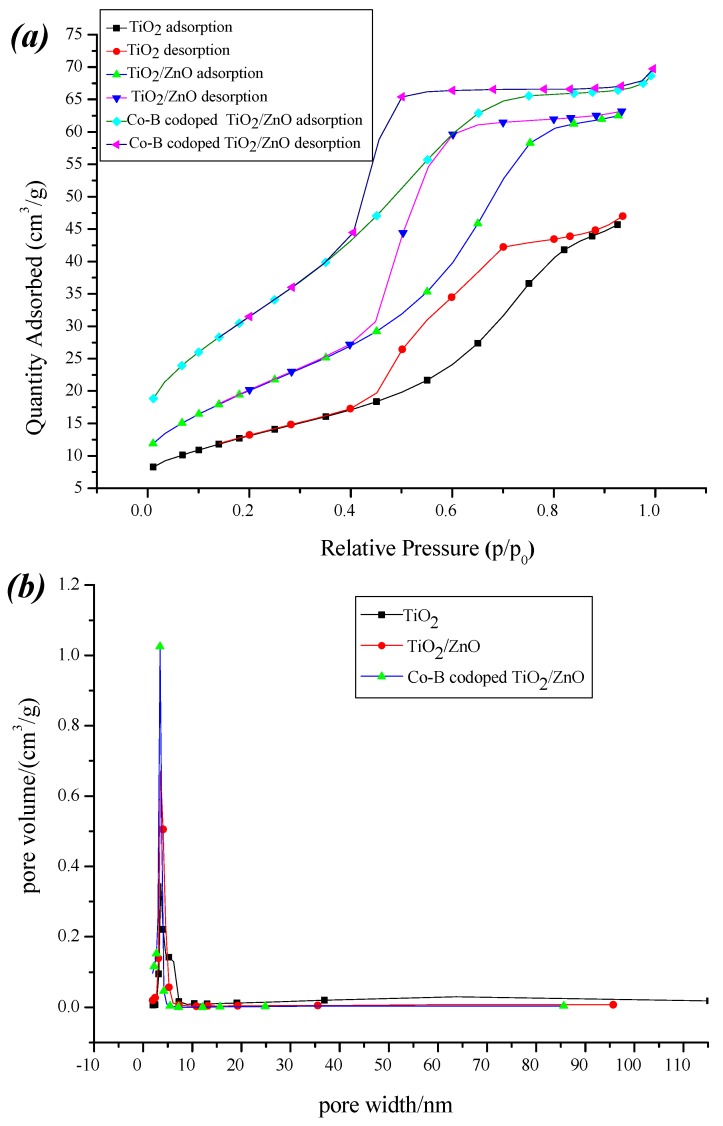
N_2_ adsorption/desorption isotherms (**a**) and pore size distribution (**b**) of pure TiO_2_, composite TiO_2_/ZnO, and Co-B codoped TiO_2_/ZnO powders.

Specific surface areas play an important role in influencing the photocatalytic activity of TiO_2_. Figure 10 shows N_2_ adsorption/desorption isotherms (a) and pore size distribution (b) of pure TiO_2_, composite TiO_2_/ZnO, and Co-B codoped TiO_2_/ZnO powders. From Figure 10a, the isotherms of the samples exhibit distinct hysteresis loops from 0.4 to 1.0 at high relative pressures. The distinct hysteresis loop is indicative of a bottle-neck mesoporous structure existed, possibly caused by non-uniform pore size. Compared with pure TiO_2_ and composite TiO_2_/ZnO, the Co-B codoped TiO_2_/ZnO sample has largest adsorption quantities at the same relative pressure. The surface area of pure TiO_2_, composite TiO_2_/ZnO, and Co-B codoped TiO_2_/ZnO are 47.8, 74.6, and 115.8 m^2^/g, respectively. The three samples have a similar narrow pore size distribution, but their pore volumes are different as shown in Figure 10b. In our case, the pore volumes are 0.077, 0.092, and 0.104 cm^3^/g for pure TiO_2_, TiO_2_/ZnO, and Co-B codoped TiO_2_/ZnO samples, respectively. The large surface area and pore volume can effectively adsorb more H_2_O, O_2_, and pollutants on the reactive sites of the catalysts, thus improving the photocatalytic activity of the modified TiO_2_.

Based on the above experiments, the Co-B codoped TiO_2_/ZnO composite films exhibit excellent photocatalytic activity under visible light irradiations. According to the FE-SEM, BET, and XRD analyses, Co-B codoping and ZnO coupling can effectively lessen the aggregation of the TiO_2_ nanoparticles, increase specific surface area of the TiO_2_ film, and inhibit the transformation of anatase to rutile at high temperature. According to the PL and DRS spectra analyses, Co-B codoping and ZnO coupling can not only induce strong visible light absorption but also reduce the recombination rate of electron–hole pairs. These factors lead to effective photodegradation of organic pollutants.

## 4. Conclusions

In summary, the Co-B codoped TiO_2_/ZnO films were successfully fabricated on the surface of common glass substrates by a simple sol-gel approach and a controllable dip-coating technique. Compared with pure TiO_2_, Co-B codoped TiO_2_/ZnO film showed excellent photocatalytic activity under visible light irradiation. According to PL, DRS, XRD, SEM, and BET analyses, the Co-B codoped TiO_2_/ZnO film with large surface area and narrowed band gap energy was composed of smaller nanoparticles compared to pure TiO_2_. The high photocatalytic performance and low cost of the Co-B codoped TiO_2_/ZnO film will make it a promising material in the application of disposing wastewater.
